# Symptomatic and asymptomatic carotid near-occlusions have very similar angiographic appearance on CT-angiography

**DOI:** 10.1007/s00234-022-03054-0

**Published:** 2022-09-21

**Authors:** Elisa Kellomäki, Thomas Gu, Allan J. Fox, Elias Johansson

**Affiliations:** 1 Clinical Science, Neurosciences, Umeå University, Umeå, Sweden; 2 Sunnybrook Health Science Centre, University of Toronto, Toronto, ON Canada; 3 Wallenberg Centre for Molecular Medicine, Umeå University, Umeå, Sweden

**Keywords:** CT-angiography, Carotid stenosis, Carotid near-occlusion, Stroke

## Abstract

The aim of this study was to compare the angiographic appearance of symptomatic and asymptomatic carotid near-occlusion. We have found no such previous study. The study hypothesis was that among symptomatic patients with ≥ 50% carotid stenosis, near-occlusion is more common and near-occlusions are more severe than among asymptomatic persons with ≥ 50% carotid stenosis. We reassessed consecutive CTAs from 4042 persons, 645 had ≥ 50% carotid stenosis, and 385 (60%) symptomatic. Near-occlusion was similarly common in symptomatic (105, 27%) and asymptomatic (56, 24%) cases. Among near-occlusions, the angiographic appearance was very similar between symptomatic and asymptomatic cases: mean stenosis lumen diameter (0.7 mm), distal ICA diameter (2.1 mm), and ECA ratio (0.79) were the same in both groups. Mean ICA ratio (0.46 and 0.48) and share of full collapse was very similar (45% and 42%). These findings add to the pathophysiological understanding of carotid near-occlusion.

## Introduction

Carotid near-occlusion is a severe carotid stenosis that causes a size reduction (collapse) of the ICA beyond the stenosis [1]. Near-occlusions can be subdivided into a less severe subset (without full collapse) and a more severe subset (with full collapse) [1]. A prognosis-based definition of full collapse (distal ICA diameter ≤ 2.0 mm and/or ICA ratio ≤ 0.42) has been proposed, resulting in a high risk of early stroke recurrence (31% within 14 days of presenting event) for full collapse and low risk (4%) for without full collapse [2]. We have found no study that compared angiographic appearances of symptomatic and asymptomatic near-occlusions. The aim of this study was to compare the angiographic appearance of symptomatic and asymptomatic near-occlusions. The study hypothesis was that among symptomatic patients with ≥ 50% carotid stenosis, near-occlusion is more common, and near-occlusions are more severe (smaller residual stenosis lumen and distal ICA) than among asymptomatic persons with ≥ 50% carotid stenosis.

## Methods

We reassessed consecutive CTA exams from 4042 persons aged ≥ 18 years from 2010 to 2014. We included all persons with extracranial ≥ 50% carotid stenosis. We reviewed the medical records and defined symptomatic as cases with an ischemic cerebrovascular event fitting with the distribution of a stenosed artery within 6 months of the exam. Three observers (EK, TG, EJ) reviewed the medical records blinded to degree of stenosis, but side of stenosis was known as this was needed for data synthesis. All cases with unclear symptomatic status were assessed by a neurologist with extensive carotid stenosis experience (EJ). Diagnosis of near-occlusion was done as described elsewhere by two observers (EJ (2 years experience), and AJF (> 40 years experience)), blinded to each other and to clinical data and with inter-rater observer kappa of 0.80 [3]. Disagreements were resolved by discussion. We used feature interpretation, diagnosing near-occlusion when the distal ICA was collapsed, and a severe proximal stenosis was the most reasonable cause. Care was taken to not mistake anatomical variation with coinciding stenosis for near-occlusion [4]. One observer (EJ) performed diameter measurements of the stenosis lumen, distal ICA (well beyond the stenosis), and ECA (just before its terminal bifurcation behind the jaw). Calipers were systematically placed in the middle of the “fuzzy edge,” without extra magnification. Full collapse was defined as distal ICA diameter ≤ 2.0 mm and/or ICA ratio ≤ 0.42 [2].

Index side was defined as the symptomatic side for symptomatic patients and as the side with most severe stenosis for asymptomatic persons. Where appropriate, we used mean, standard deviation, median, and interquartile range. We used *t*-test and *χ*^2^-test in bivariate analyses. In multivariable analyses (linear and binary logistic regression), we used all parameters with *p* ≤ 0.1 in the baseline assessment as co-variates. *p* < 0.05 was considered statistically significant. A Bonferroni-corrected *p*-value threshold was calculated as 0.05/m for the two analyses with multiple testing (Tables 1 and 2), where “*m*” is number of tests. IBM SPSS 28.0 was used for calculations.

## Results

We included 645 participants with ≥ 50% carotid stenosis, of which 385 (60%) were symptomatic. There were several baseline differences between the symptom groups, albeit some might have been false positive due to multiple testing (Table 1). Comparing symptomatic and asymptomatic cases, there were no differences in share of degree of the three stenosis groups (bivariate *p* = 0.23, Table 1; multivariable *p* = 0.38, data not shown), nor between near-occlusion and conventional ≥ 50% stenosis (bivariate analyses *p* = 0.12, Table 1; multivariable *p* = 0.28, data not shown). Among near-occlusions, there were no differences in any parameter of how severe the near-occlusion was between symptomatic and asymptomatic cases in bivariate and multivariable analyses (Table 2).

**Table 1:** Baseline characteristics and CTA findings in the entire study population

		Asymptomatic (*n* = 260)	Symptomatic (*n* = 385)	*p*^a^
Age mean (SD)		72 (8)	72 (8)	0.84
Women *n* (%)		99 (38)	118 (31)	0.051
Previous myocardial infarction n (%)^b^		61 (24)	70 (18)	0.11
Current angina *n* (%)^b^		49 (19)	55 (14)	0.13
Current heart failure *n* (%)^b^		22 (8)	24 (6)	0.35
Current claudication *n* (%)^b^		23 (9)	23 (6)	0.21
Previous arterial revascularization *n* (%)^b^		91 (35)	67 (17)	** < 0.001**
Atrial fibrillation *n* (%)^b^		39 (15)	37 (10)	0.046
Current smoking *n* (%)^b^		51 (20)	70 (18)	0.68
Hypertension *n* (%)^b,c^		246 (95)	345 (90)	0.011
Diabetes *n* (%)^b^		60 (23)	95 (25)	0.71
Total cholesterol mean (SD)^d^		4.8 (1.3)	4.9 (1.3)	0.28
LDL cholesterol mean (SD)^d^		2.7 (1.1)	2.9 (1.3)	0.024
HDL cholesterol mean (SD)^d^		1.28 (0.40)	1.19 (0.31)	0.007
Referred from other hospital *n* (%)		160 (62)	290 (75)	** < 0.001**
Presenting event	Stroke *n* (%)	NA	197 (51)	**–**
	TIA *n* (%)	NA	136 (35)	
	Retinal *n* (%)	NA	52 (14)	
Delay presenting event to CTA median (IQR)		NA	3 (0–6)	**–**
CTA indication	Cerebrovascular event, other cause *n* (%)^e^	125 (48)	NA	**–**
	Suspected cerebrovascular event *n* (%)^f^	72 (28)	NA	
	Other *n* (%)^g^	63 (24)	NA	
CTA findings on index side	Conventional stenosis *n* (%)	204 (78)	280 (73)	0.24^ h^
	Near-occlusion without full collapse *n* (%)	31 (12)	61 (16)	
	Near-occlusion with full collapse *n* (%)	25 (10)	44 (11)	
	Degree of conventional stenosis mean (SD)	64 (9)	68 (10)	** < 0.001**
≥ 50% stenosis or occlusion on contralateral side *n* (%)		73 (28)	112 (29)	0.79

*HDL* high-density lipoprotein, *IQR* interquartile range, *LDL* low-density lipoprotein, *NA* not applicable, *SD* standard deviation, *TIA* transient ischemic attack

^a^2-sided tests: *T*-test for continuous variables and *χ*^2^-test for categorical variables. Bonferroni-corrected threshold after adjustment for multiple testing was *p* < 0.0028. Significant findings after correction highlighted in bold

^b^Missing data in 1–2 cases

^c^Defined as blood pressure > 140/90 and/or use of blood pressure reducing medication

^d^Missing data in 104–111 cases

^e^73 contralateral ischemic event with < 50% stenosis or occlusion on the symptomatic side, 41 posterior circulation ischemia (PCA territory considered anterior circulation in cases with fetal PCA), 7 hemorrhage, and 4 ipsilateral ischemic events but clearly due to other causes (multiple territory fresh ischemia *n* = 3 and iatrogenic stroke during neck-dissection surgery *n* = 1)

^f^CTA was performed as part of cerebrovascular work-up, but final diagnosis was not cerebrovascular disease (such as syncope and seizure)

^g^25 follow-up of known stenosis, 23 Carotid bruit, 11 Other diseases (such as head-neck cancer) and 4 research exams

^h^Near-occlusion compared to conventional stenosis had *p* = 0.12. Near-occlusion with full collapse compared to all other degree of stenosis had *p* = 0.52

**Table 2:** CTA findings among cases with near-occlusion

	Asymptomatic (*n* = 56)	Symptomatic (*n* = 105)	Bivariate *p*^a^	Multivariable *p*^b^
Stenosis diameter mm mean (SD)^c^	0.7 (0.2)	0.7 (0.2)	0.75	0.59
Reduced opacity in stenosis n (%)^c,d^	25 (49)	48 (49)	1.0	0.27
Distal ICA diameter mm mean (SD)	2.1 (1.1)	2.1 (1.0)	0.86	0.29
ICA ratio mean (SD)^e^	0.46 (0.24)	0.48 (0.25)	0.63	0.71
ECA ratio mean (SD)	0.79 (0.49)	0.79 (0.51)	0.99	0.63
Full collapse *n* (%)	25 (45)	44 (42)	0.87	0.35
≥ 50% stenosis or occlusion on contralateral side *n* (%)	21 (38)	32 (30)	0.38	0.67

ECA ratio ipsilateral ICA/ECA

*ICA* internal carotid artery, *ICA ratio* ipsilateral/contralateral distal ICA

^a^2-sided tests: *T*-test for continuous variables and *χ*^2^-test for categorical variables. Bonferroni-corrected threshold after adjustment for multiple testing was *p* < 0.0071

^b^Linear logistic regression for continuous variables and binary logistic regression for categorical variables. Controlled for all variables with *p* ≤ 0.1 in baseline assessment (sex, revascularization, atrial fibrillation, hypertension, LDL, HDL, and referred)

^c^Missing data in 12 cases due to extensive calcification

^d^In tiny stenosis lumen, contrast was darker than surrounding contrast. Presumably due to partial volume effect. Arbitrarily assigned to have a 0.5-mm diameter in analyses

^e^Missing data in 8 cases due to contralateral occlusion

## Discussion

The main finding of this study was that there was no difference in angiographic appearance of near-occlusions between symptomatic patients and asymptomatic persons.

The mechanism of the recurrent strokes in near-occlusion with full collapse is unknown. Cerebral hypoperfusion has been proposed [5–7]. As ICA diameter is closely linked to ICA flow [8], full collapse causes a large blood flow reduction through the ICA. However, the autoregulation system will recruit collaterals by dilation of cerebral arterioli. Reasonably, less dilatation will be required in case with good collateral capacity and vice-versa. The amount of dilation capacity remaining (cerebrovascular reserve) can be assessed with TCD and SPECT. No remaining (“exhausted”) reserve is a marker of cerebral hypoperfusion. In a SPECT study with 15 near-occlusions, some near-occlusions had exhausted cerebrovascular reserve, some just reduced, but the analysis did not distinguish between symptomatic and asymptomatic near-occlusion or between with and without full collapse [5]. In a TCD study, with 50 symptomatic near-occlusions, 22 (44%) had reduced and 11 (22%) had exhausted cerebrovascular reserve, with similar findings for with and without full collapse [6]. In contrast, Greiner et al. briefly reported that among 53 near-occlusions (seemingly all with full collapse), the cerebrovascular reserve was always reduced, but never exhausted [9]. Near-occlusions often have collateral recruitment [7], why some degree of dilation is expected. However, if the mechanism of stroke in symptomatic full collapse is hypoperfusion, many such cases should have exhausted cerebrovascular reserve, clearly more often than both asymptomatic near-occlusion with full collapse and symptomatic near-occlusion without full collapse. However, this has never been shown. If anything, the opposite has been shown [6, 9]. To add to this, we report no difference in angiographic appearance between symptomatic and asymptomatic near-occlusion. If cerebral hypoperfusion was the mechanism, it would be reasonable that when a stenosis progresses to full collapse, there would be a selection with some cases becoming symptomatic at that point. If so, there should be relatively few asymptomatic near-occlusions with full collapse — which we refute. This was not caused by chance due to an unfortunate threshold for full collapse as we also found no difference in near-occlusion severity when assessed on a spectrum (distal ICA diameter and ICA ratio). Thus, our study adds to the growing body of evidence that the mechanism of stroke in near-occlusion with full collapse is not cerebral hypoperfusion. Further studies are warranted. These should also include assessment of embolic mechanism as artery ligation has been proposed as a possible treatment for near-occlusion with full collapse if the mechanism is embolic [10].

Strengths of this study were the large sample size and rigorous classification of symptom status and degree of stenosis. Weaknesses include lack of test of cerebrovascular reserve and possible selection bias among asymptomatic stenoses, as many were detected by cerebrovascular disease and more symptomatic patients came by referral than asymptomatic persons.

In summary, there is no difference in angiographic appearance between symptomatic and asymptomatic carotid near-occlusions. These findings add to the pathophysiological understanding of carotid near-occlusion.
